# Full-endoscopic Removal of a Lumbar Synovial Cyst after Laminectomy for Lumbar Spinal Canal Stenosis

**DOI:** 10.31662/jmaj.2024-0394

**Published:** 2025-05-30

**Authors:** Tatsuya Maegawa, Eitaro Okumura, Kotaro Kohara, Ryo Hashimoto, Motoo Kubota

**Affiliations:** 1 Department of Spinal Surgery, Kameda Medical Center, Kamogawa, Japan

**Keywords:** full-endoscopic laminectomy, full-endoscopic spinal surgery, synovial cyst

An 84-year-old man with a history of percutaneous coronary intervention, anticoagulation therapy for atrial fibrillation, and corticosteroid use for nephrotic syndrome presented with recurrent lumbar spinal stenosis. Two years earlier, he had undergone decompression surgery at L3/4/5/S1, followed by a repeat procedure one year later owing to recurrence. Despite initial relief, intermittent claudication returned. Magnetic resonance imaging (MRI) and computed tomography (CT) revealed re-stenosis at L3/4 caused by a synovial cyst (Figure 1). Considering his comorbidities, minimally invasive full-endoscopic spine surgery (FESS) was performed. Intraoperatively, the synovial cyst with respiratory pulsations was excised through additional fenestration (Figure 2). Postoperative MRI and CT confirmed adequate decompression, and the patient’s symptoms resolved, achieving an excellent outcome per MacNab criteria (Figure 3).

**Figure 1.**
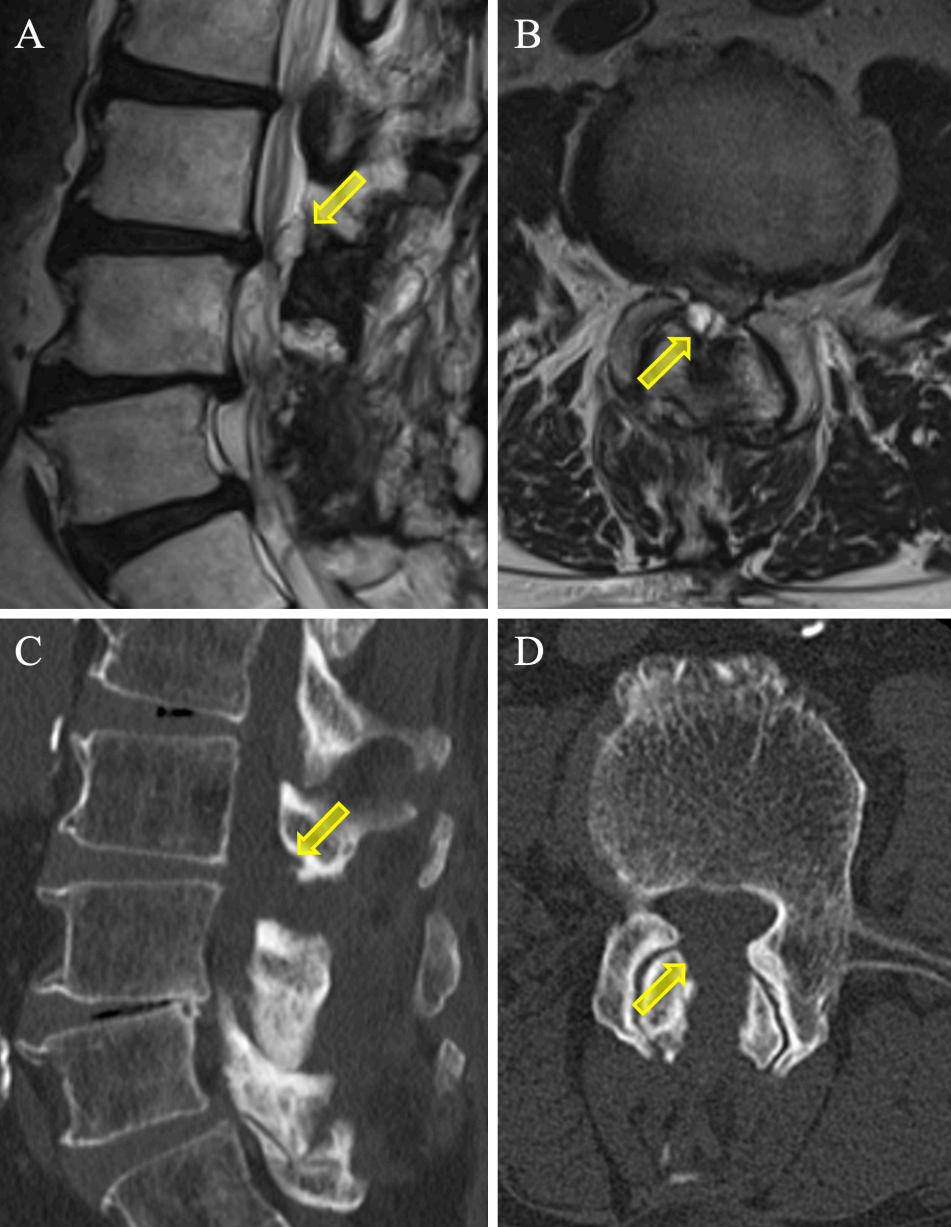
Preoperative T2-weighted MRI images (A, sagittal; B, axial) and CT scans (C, sagittal; D, axial) showing the dural sac severely compressed by a synovial cyst from the right facet at the L3/4 level (*arrow,* synovial cyst).

**Figure 2.**
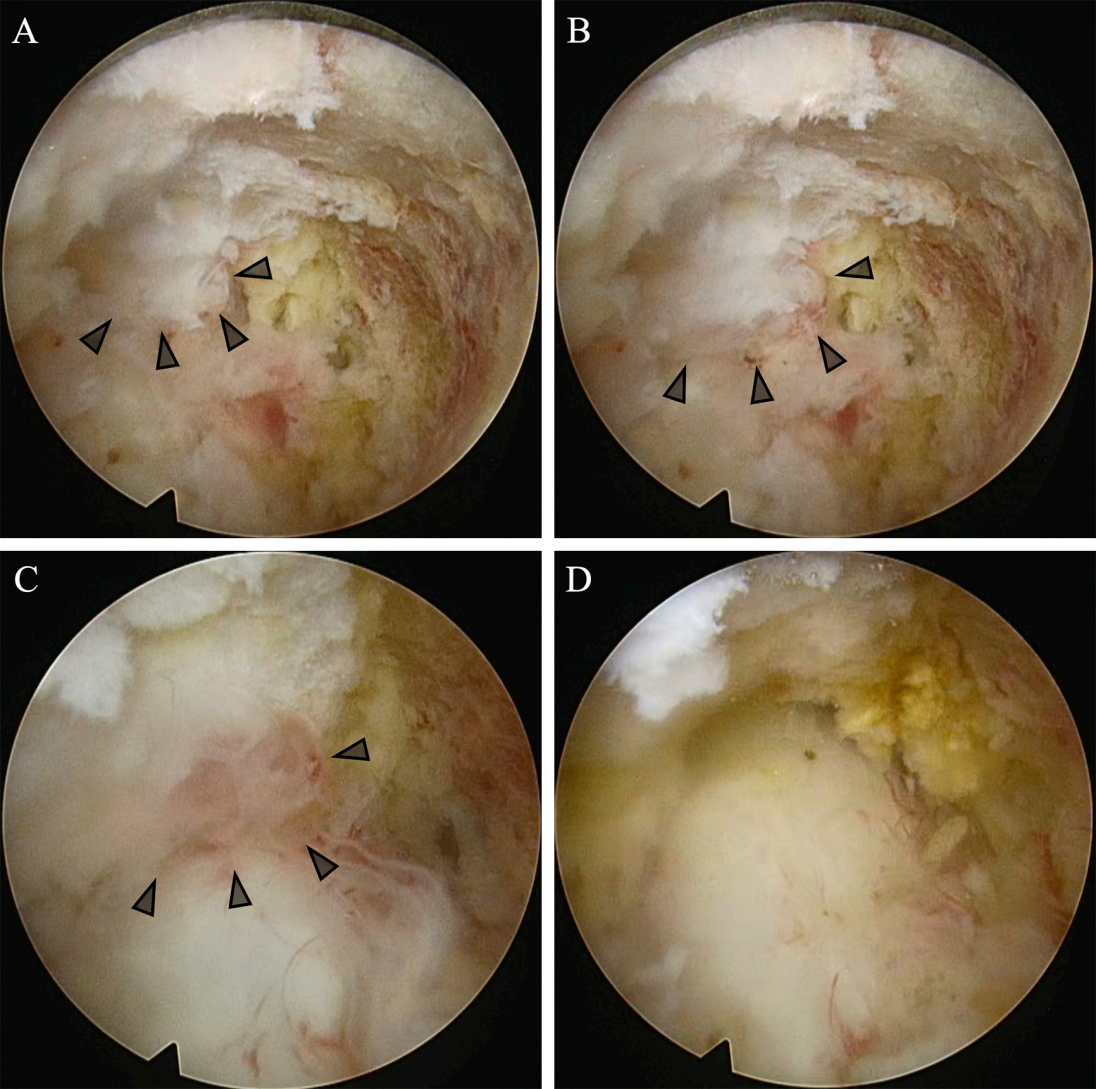
Intraoperative view with full-endoscopic spine surgery (FESS). The synovial cyst repeatedly shrank (A) and swelled (B) on the basis of respiratory fluctuations. The view (C) just before resection of the synovial cyst, and its removal, causing good pulsation of the dural sac (D) (*arrowhead*; synovial cyst).

**Figure 3.**
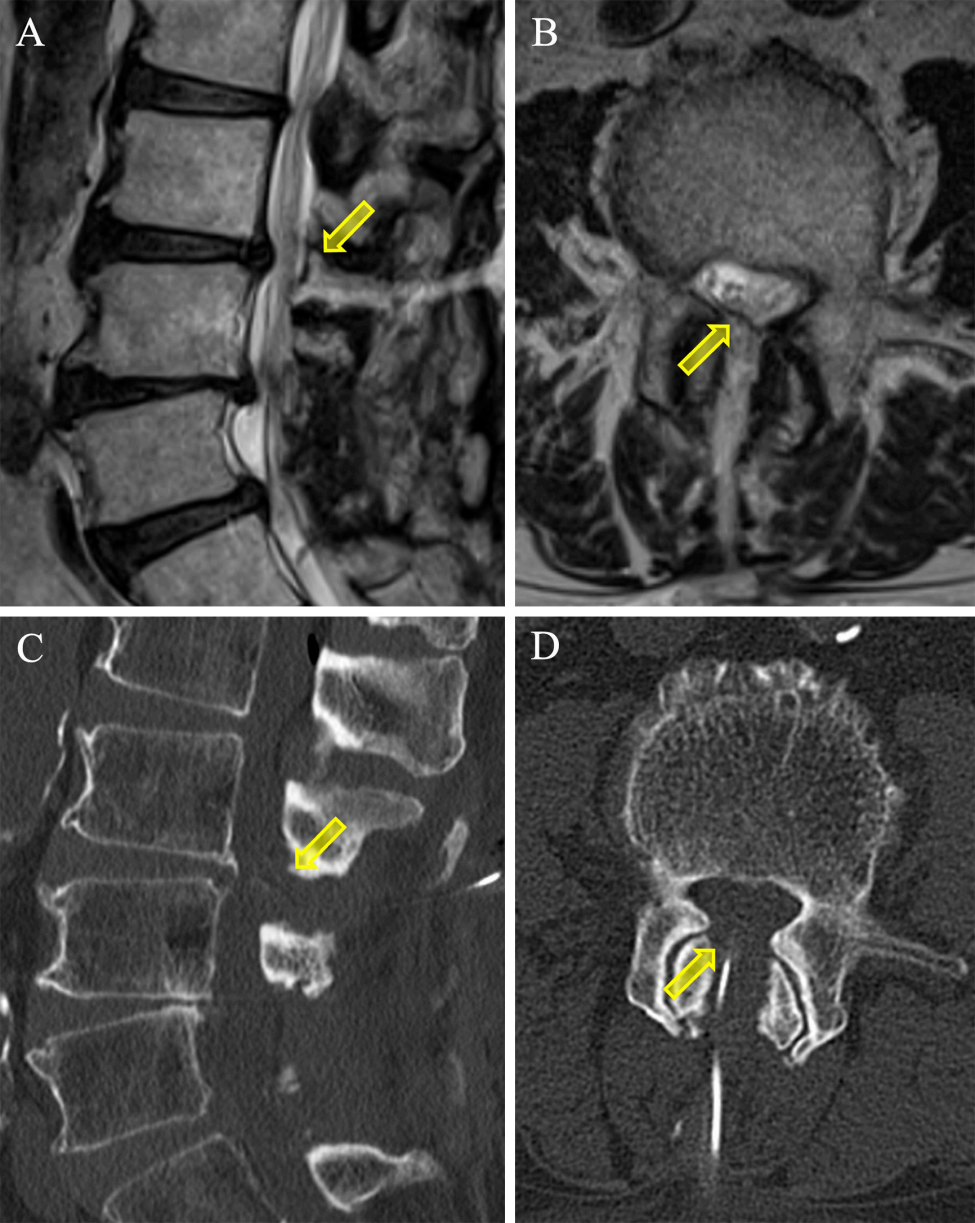
Postoperative T2-weighted MRI images (A; sagittal, B; axial) and CT scans (C; sagittal, D; axial) showing adequate decompression of the dural sac at the L3/4 level (*arrow*; disappearance of synovial cyst). CT: computed tomography; MRI: magnetic resonance imaging.

Synovial cysts, often associated with instability, degenerative spondylolisthesis, and facet joint arthritis, are rare, comprising 5% of cases ^(1), (2), (3)^. To the best of our knowledge, this is the first reported case of FESS for a postoperative synovial cyst, indicating its safety and potential for wider clinical application ^(3), (4), (5)^.

## Article Information

### Conflicts of Interest

None

### Acknowledgement

We thank Editage (www.editage.jp) for English language editing.

### Approval by Institutional Review Board (IRB)

23-005-240411, institutional review board in Kameda Medical Center.

### Ethical Considerations

When reporting these cases, care was taken to protect the patient’s personal information and privacy, and consent was obtained from the patient for publication of the details contained herein.

### Informed Consent

Written informed consent was obtained from the patient to publish this case, including pictures.
